# Gene therapies development: slow progress and promising prospect

**DOI:** 10.1080/20016689.2017.1265293

**Published:** 2017-01-03

**Authors:** Eve Hanna, Cécile Rémuzat, Pascal Auquier, Mondher Toumi

**Affiliations:** ^a^ Public Health Department, Aix Marseille University, Paris, France; ^b^ Creativ-ceutical, Paris, France

**Keywords:** Gene therapy, clinical trials, market access

## Abstract

**Background**: In 1989, the concept of human gene therapies has emerged with the first approved human gene therapy trial of Rosenberg et al. Gene therapies are considered as promising therapies applicable to a broad range of diseases.

**Objective**: The objective of this study was to review the descriptive data on gene therapy clinical trials conducted worldwide between 1989 and 2015, and to discuss potential success rates of these trials over time and anticipated market launch in the upcoming years.

**Methods:** A publicly available database, ‘Gene Therapy Clinical Trials Worldwide’, was used to extract descriptive data on gene therapy clinical trials: (1) number of trials per year between 1989 and 2015; (2) countries; (3) diseases targeted by gene therapies; (4) vectors used for gene delivery; (5) trials status; (6) phases of development.

**Results**: Between 1989 and 2015, 2,335 gene therapy clinical trials have been completed, were ongoing or approved (but not started) worldwide. The number of clinical trials did not increase steadily over time; it reached its highest peak in 2015 (163 trials). Almost 95% of the trials were in early phases of development and 72% were ongoing. The United States undertook 67% of gene therapy clinical trials. The majority of gene therapies clinical trials identified targeted cancer diseases.

**Conclusion**: The first gene therapy was approved in the European Union in 2012, after two decades of dashed expectations. This approval boosted the investment in developing gene therapies. Regulators are creating a specific path for rapid access of those new therapies, providing hope for manufacturers, healthcare professionals, and patients. However, payers are increasingly scrutinizing the additional benefits of the new therapies. Major steps forward are expected in the field of gene therapies in the future.

## Background and objective

The concept of human gene therapies emerged in 1989, with the first approved human gene therapy trial of Rosenberg et al. [1]. This trial investigated the use of human tumour-infiltrating lymphocytes (TIL) genetically modified by introducing the gene coding for resistance to neomycin into TIL for patients with advanced melanoma. Retroviral-mediated gene transduction was used to introduce the gene coding for resistance to neomycin into human TIL before their infusion into patients. This study demonstrated the feasibility and safety of using retroviral gene transduction for human gene therapy. In addition, it demonstrated that lymphocytes may be suitable for the gene therapy of other diseases, since they persist for long periods in the circulation and the tissue.

The European Medicines Agency (EMA) defines a gene therapy medicinal product (GTMP) as a:

biological medicinal product that contains an active substance which contains or consists of a recombinant nucleic acid used in or administered to humans to regulate, repair, replace, add or delete genetic sequence and its therapeutic, prophylactic or diagnostic effect relates directly to the recombinant nucleic acid sequence it contains, or to the product of genetic expression of this sequence.

Vaccines against infectious diseases are not considered as GTMP [2]. In Europe, gene therapies are classified as Advanced Therapy Medicinal Products (ATMPs). ATMP is a class of innovative therapeutics that includes, in addition to gene therapies, cell therapies and tissue engineered products. The legal and regulatory framework for ATMPs in the European Union (EU) was established by the EU Commission in 2007 (Regulation EC No. 1394/2007) and first applied in December 2008 [3].

The Food and Drug Administration (FDA) defines gene therapy as products:

that mediate their effects by transcription and/or translation of transferred genetic material and/or by integrating into the host genome and that are administered as nucleic acids, viruses, or genetically engineered micro-organisms. The products may be used to modify cells in vivo or transferred to cells ex vivo prior to administration to the recipient [4].

Gene therapies are promising therapies applicable to a broad range of diseases; their aim is to radically treat the causes of the diseases instead of only relieving the symptoms. They may be effective on a wide range of previously untreated diseases, such as haematological, ocular, neurodegenerative diseases, and several cancers [5]. For example, Adeno-associated AAV2 vectors carrying the therapeutic gene (RPE65) intra-retinal injection resulted in improved vision for people with Leber’s Congenital Amaurosis [6,7]. Murine γ-retroviral vectors have also been employed in gene therapy trials of Adenosine deaminase deficiency (ADA-SCID), a fatal primary immunodeficiency with impaired T-, B-, and NK-cell development [8].

Gene therapies are regarded as a potential revolution in the health sciences and pharmaceutical fields. The number of clinical trials investigating gene therapies is increasing worldwide, despite the limited number of products that have successfully reached the market. Almost three decades after the first gene therapy trial, only three gene therapies were approved in EU: Glybera® (alipogene tiparvovec) [9]; Imlygic® (talimogene laherparepvec) [10]; Strimvelis® (autologous CD34+ cells transduced to express ADA) [11]; and only Imlygic® has been approved in the United States (US) [12].

Ginn et al. [13], showed that over 1,800 gene therapy clinical trials were completed, ongoing, or approved until 2012. This field is in progress with promising results. We believe that the approval of Glybera® in EU in 2012 may have fostered the development of gene therapies.

Therefore, the objective of this study was to review the descriptive data on gene therapy clinical trials conducted worldwide between 1989 and 2015, and to discuss potential success rates of these trials over time and anticipated market launch in the upcoming years.

## Methods

The data used in the article were extracted from an interactive publicly available database, ‘Gene Therapy Clinical Trials Worldwide’, provided by the journal of Gene Medicine [14]. At the time of writing, data were last updated in February 2016. The database presented information on individual gene therapy trials performed worldwide, including: trial country; principal investigator; disease category; indication; vector used; gene transferred; gene type; clinical phase; trial status; and the year trial approved/initiated. The sources of the data of this database were official agency sources (Research Administration and Compliance [RAC], Gene Therapy Advisory Committee [GTAC]), the published literature, presentations at conferences, and from information provided by investigators or trial sponsors. In this database, information on the trials performed in the US was derived directly from the Office of Biotechnology Activities [OBA]/RAC website.

We extracted data from clinical trials that started between 1989 and 2015 including: (1) number of trials per year between 1989 and 2015; (2) countries where the trials were conducted i.e., multi-country, US, United Kingdom (UK), Germany, China, France, Switzerland, Japan, The Netherlands, Australia, Canada, or others; (3) diseases targeted by gene therapies, i.e., cancer diseases, cardiovascular diseases, infectious diseases, inflammatory diseases, monogenic diseases (cystic fibrosis, Huntington’s disease, Fanconi anaemia, Gaucher disease, severe combined immunodeficiency (SCID), Haemophilia A and B, Hurler syndrome, Hunter syndrome and others), neurological diseases, ocular diseases, others; (4) vectors used for gene delivery, i.e., adeno-associated virus, adenovirus, retrovirus, vaccinia virus, lentivirus, herpes simplex virus, lipofection, naked/plasmid DNA, poxvirus, RNA transfer (see supplemental material); (5) trials status, i.e., closed, withdrawn, on clinical hold, conditional approval, cancelled, under review, submission not completed and (6) phases, i.e., I, I/II, II, II/III, III, IV, or single subject.

## Results

**Figure 1.**
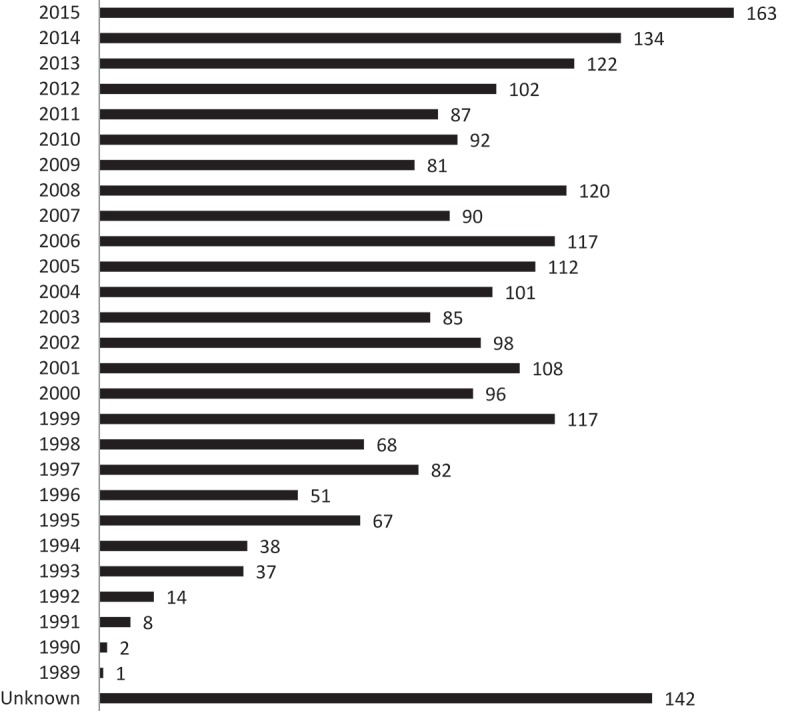
Number of gene therapy trials per year.

Between 1989 and 2015, 2,335 clinical trials related to gene therapies had been completed, were ongoing or approved (but not started) worldwide. After the first gene therapy trial in 1989, the number of clinical trials increased over time (Figure 1). However, this number did not rise steadily, but it reached a peak in 1999 (117 trials), in 2008 (120 trials), then dropped between 2009 and 2012. Since 2012, the number of clinical trials has considerably increased, to reach its highest peak in 2015 (163 trials).

**Figure 2.**
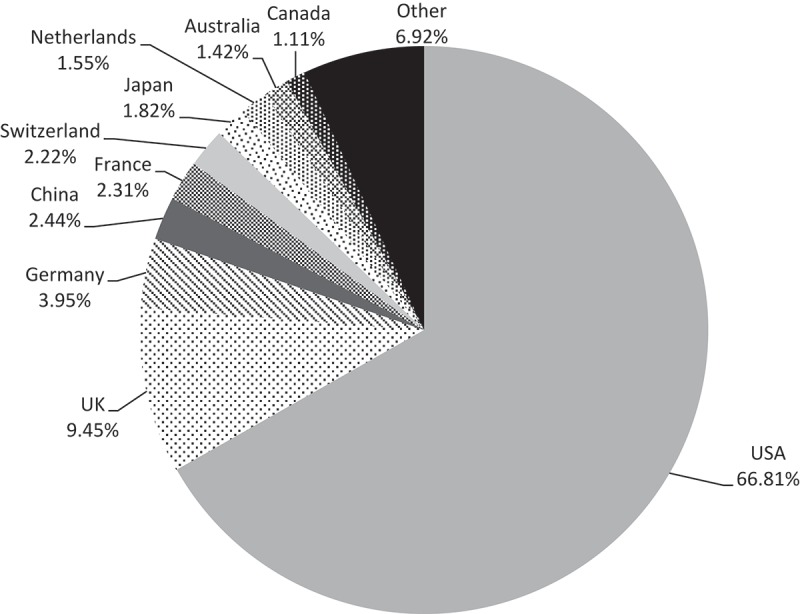
Distribution of gene therapy clinical trials by country.

The US undertook 66.81% of gene therapy clinical trials; all other countries participated in a small percentage of the trials: 9.45% in the UK; 3.95% in Germany; and around 2% each in Switzerland, France, China, and Japan (Figure 2).

**Figure 3.**
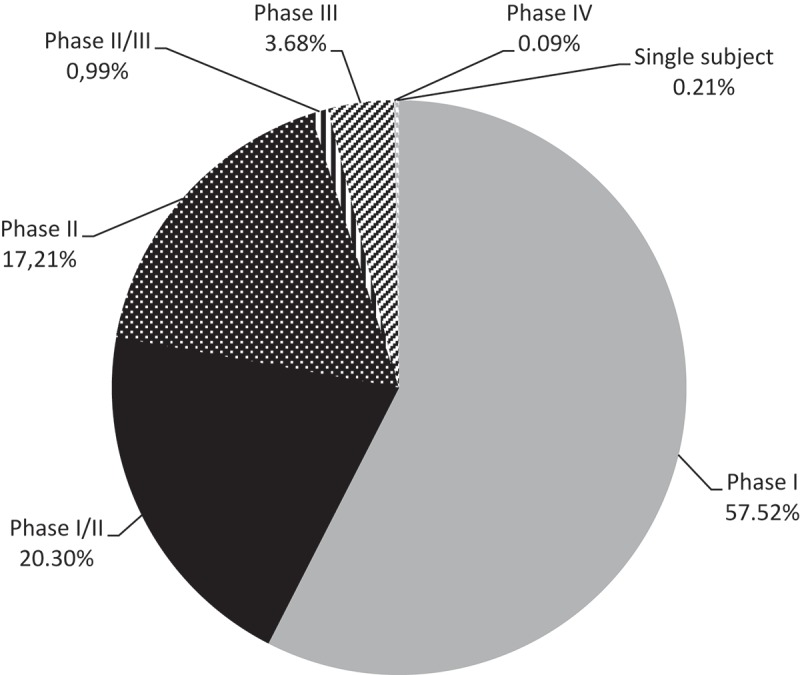
Phase of development of gene therapy clinical trials.

Almost 95% of the trials were in early phases of development; 57.52% of the trials were Phase I trials, 20.30% were Phase I/II, and 17.21% Phase II. Gene therapies in phase II/III, III and IV constituted only 5% of the trials (Figure 3).

Seventy-two per cent of the trials were ongoing, 24.83% were closed, and 1.20% of the trials were withdrawn. The withdrawn trials were related to products in early phases of development. Seventy-one Phase III trials (82.5% of Phase III trials) were ongoing, 11 trials were closed, 2 were cancelled, 1 was under review, and 1 submission was not completed. The two Phase IV trials were ongoing (Table 1).

**Table 1.** Phase and status of gene therapy clinical trials.

	Phase I	Phase I/II	Phase II	Phase II/III	Phase III	Phase IV	Single subject	Total
Open	970	327	288	15	71	2	5	1,678
	(57.81%)	(19.49%)	(17.16%)	(0.89%)	(4.23%)	(0.12%)	(0.30%)	(71.86%)
Closed	337	129	96	7	11	–	–	580
	(58.10%)	(22.24%)	(16.55%)	(1.21%)	(1.90%)			(24.83%)
Withdrawn	12	8	8	–	–	–	–	28
	(42.86%)	(28.57%)	(28.57%)					(1.20%)
On clinical hold	3	3	1	–	–	–	–	7
	(42.86%)	(42.86%)	(14.28%)					(0.30%)
Conditional approval	9	1	5	–	–	–	–	15
	(60.00%)	(6.67%)	(33.33%)					(0.64%)
Canceled	–	2	1	1	2	–	–	6
		(33.33%)	(16.67%)	(16.67%)	(33.33%)			(0.26%)
Under review	10	3	1	–	1	–	–	15
	(66.67%)	(20.00%)	(6.67%)		(6.67%)			(0.64%)
Submission not	2	1	2	–	1	–	–	6
completed	(33.33%)	(16.67%)	(33.33%)		(16.67%)			(0.26%)
Total	1,343	474	402	23	86	2	5	2,335
	(57.52%)	(20.30%)	(17.21%)	(0.99%)	(3.68%)	(0.09%)	(0.21%)	(100.00%)

The majority of gene therapy clinical trials targeted cancer diseases (64.41%). 52% of Phase II/III trials, 66% of the Phase III trials and all the Phase IV trials were for gene therapies targeting cancers (Table 2).

**Table 2.** Number of gene therapy clinical trials by phase and indication.

	Cancer diseases	Cardiovascular diseases	Gene marking	Healthy volunteers	Infectious diseases	Inflammatory diseases	Monogenic diseases	Neurological diseases	Ocular diseases	Others	Total
Phase I	886	76 (5.65%)	42	41	106	9	128	16	14	25	1,343
	(65.97%)		(3.12%)	(3.05%)	(7.89%)	(0.68%)	(9.54%)	(1.19%)	(1.04%)	(1.86%)	(57.51%)
Phase	273	34	5	2	44	–	78	15	10	13	474
I/II	(57.59%)	(7.17%)	(1.06%)	(0.43%)	(9.28%)		(16.45%)	(3.16%)	(2.11%)	(2.74%)	(20.29%)
Phase II	271	50	3	8	22	5	13	12	8	10	402
	(67.41%)	(12.44%)	(0.75%)	(1.99%)	(5.47%)	(1.24%)	(3.23%)	(2.98%)	(1.99%)	(2.49%)	(17.22%)
Phase	12	7	–	–	–	–	4	–	–	–	23
II/III	(52.17%)	(30.43%)					(17.40%)				(0.98%)
Phase III	57	10	–	2	6	–	6	–	1	4	86
	(66.28%)	(11.63%)		(2.32%)	(6.98%)		(6.98%)		(1.16%)	(4.65%)	(3.68%)
Phase	2	–	–	–	–	–	–	–	–	–	2
IV	(100%)										(0.08%)
Single	3	–	–	–	–	–	2	–	–	–	5
subject	(60.00%)						(40.00%)				(0.21%)
Total	1504	177	50	53	178	14	231	43	33	52	2,335
	(64.41%)	(7.58%)	(2.14%)	(2.27%)	(7.62%)	(0.60%)	(9.90%)	(1.84%)	(1.41%)	(2.22%)	(100.00%)

Monogenic diseases constituted the indication of 9.90% of the trials encompassing cystic fibrosis, Huntington’s disease, Fanconi anaemia, Gaucher disease. Almost 8% of the trials targeted each of cardiovascular and infectious diseases.

Adenovirus, retrovirus, and naked/plasmid DNA were the most used vectors in the gene therapy trials, with respectively 22.14%, 18.76%, and 18.03% of the trials. Adeno-associated virus vectors were used in 6.63% of the trials, and vaccinia virus, lentivirus, and lipofection were used as vectors in around 5% of the trials (Table 3).

**Table 3.** Vectors used for gene delivery.

Vectors	Number (%)
Adenovirus	517 (22.14%)
Retrovirus	438 (18.76%)
Naked/plasmid DNA	421 (18.03%)
Adeno-associated virus	155 (6.63%)
Lentivirus	134 (5.73%)
Vaccinia virus	123 (5.23%)
Lipofection	115 (4.92%)
Poxvirus	103 (4.41%)
Herpes simplex virus	84 (3.59%)
RNA transfer	43 (1.84%)
Unknown	75 (3.21%)
Others	127 (5.44%)

## Discussion

### Number of trials approved/initiated per year

The first gene therapy clinical trial was performed by Rosenberg et al. [1]; since then, companies started to invest increasingly in the development of these therapies, and the number of gene therapy trials started to rise. However, this number did not increase steadily. The drop-offs periods were the consequences of the publication of some reports on gene therapies’ adverse events [15–19]. However, between 2012 and 2015, we noticed a prominent increase in the number of trials. Indeed, in 2012, Glybera® (alipogene tiparvovec) was the first gene therapy approved in EU for the treatment of adult patients diagnosed with familial lipoprotein lipase deficiency (LPLD) confirmed by genetic testing [20,21], and suffering from severe or multiple pancreatitis attacks despite dietary fat restrictions. This therapy was granted a European orphan drug designation in March 2004, and was approved in EU under exceptional circumstances. Exceptional circumstances procedure was granted due to the rarity of the disease; it has not been possible to obtain complete information about the medicine; every year, EMA will review any new information that becomes available to reassess the risk-benefit balance [22]. The green light given to this first gene therapy may have been a turning point that led to investors’ enthusiasm for the development of gene therapies. This may had stimulated pharmaceutical companies to invest more in the development of gene therapies, as reflected in the increasing number of clinical trials between 2012 and 2015 (521 trials between 2012 and 2015).

Coherently, a fourfold increase in the number of gene therapies since 2012 has been shown in a survey conducted in November 2015 [23]; the gene therapy products in development from preclinical phase to Phase III and beyond worldwide identified were 418 products.

### Countries where gene therapy clinical trials were conducted

Gene therapy clinical trials were performed in 36 countries from the 5 continents. Four per cent of these trials were performed in more than one country at the same time. The geographical distribution of trials had slightly changed from 2012 to 2015; America’s part had slightly increased to 68% (65.1% in 2012), whereas the European part had slightly decreased to 24%, instead of 28.3% in 2012. As in 2012, the US undertook the majority of the trials (66.81%), and the UK was leading almost half of European gene therapy trials. A slow growth was observed in Asia, China reached 2.44% of the trials (1.4% in 2012), and Japan 1.82% (1.1% in 2012).

These data confirm the leading role of the US in pharmaceutical innovation [24]. Actually, it was widely agreed that the US dominated the pharmaceutical innovation for decades. This is driven by a friendly environment characterized by the possibility to raise capital [25] (through angled business investors up to large investment organisation), the high funding level for health science research with organisations like the US National Institutes of Health (NIH) [26] and private foundations and organisations [27], the broad experience in university/private research contracting, as well as easiness of academic spin off [28], and finally, a favourable tax scheme for research investment [29]. After the US, the UK offers a similar friendly environment for entrepreneurs by offering research and development tax relief [30]. It is much less the case for France and Germany; while research performance may not be inferior in those countries but may be less oriented toward research private valorisation and value development.

### Diseases targeted by gene therapies

Cancer was the most common indication for gene therapies in development (64.41%), including different types of cancer: gynaecological; nervous system; gastrointestinal; genitourinary; skin; head and neck; lung; mesothelioma; haematological; and sarcoma. Due to the widespread incidence of cancer that is increasing steadily, and the important medical needs in this field, manufacturers are incentivised to invest in the field of oncology; the majority of the clinical trials in advanced phase of development are for gene therapies aiming to treat several cancers. Oncology represents a very attractive field for pharmaceutical companies, as payers have shown a very high willingness to pay, including for minor improvement, allowing a fast return on investment. Therefore, oncology has become by far the first target for drugs in development, including for small molecules and targeted therapies. According to the Pharmaceutical Research and Manufacturers of America (PhRMA), 771 new oncology drugs and vaccines are currently in clinical trials or have been submitted to the FDA for review in US companies [31]. Pharmaceutical company investments remain high, and cancer therapies account for more than 30% of all preclinical and Phase 1 clinical development [32].

The second most popular indication for gene therapies was monogenic diseases; it was targeted by 10% of all the gene therapy trials. This is not surprising, as those diseases are related to one single gene defect and gene therapies are potentially able to correct the gene defect [33]. Moreover, as rare conditions, those diseases are expected to reach the market with fewer requirements than common diseases and high prices [34]. They (or their developers) are granted special incentives, such as national tax grants or exemption from ‘across the board’ price cuts or taxes [35,36]. This makes monogenic diseases an attractive target for manufacturers and investors.

### Clinical trials phase and status for gene therapies

Coherently with our previous research on ATMPs [37], we showed that majority of the gene therapy trials were in early phases of development. This fact may be related to the approval of the first gene therapy, Glybera®, in the EU in 2012. This step encouraged investment of manufacturers in this field and fostered the development of gene therapies. As it was explained by Thierry van den Driessche, former president of the European Society for Cell and Gene Therapy and current Head of the Division of Gene Therapy and Regenerative Medicine at the Free University of Brussels, Belgium, ‘It sets a precedent for future gene therapy development and I hope it will foster collaboration between academia and industry and help to catalyse industry-driven product development.’ [38] The fact that most of the trials are in early phase suggests a wave of gene therapies filing for regulatory approvals within a 5–10-year period, if these therapy prove their clinical promises. This will raise the question of funding and impact on health insurance sustainability and patient’s access. More thoughts on this topic should be considered, as it represents on one side a threat for health insurance sustainability, and also a potential threat for return on investment if these innovations precipitate down the payers’ willingness to pay.

### Gene types and vectors used for gene therapy

Different vector systems are used nowadays for gene delivery; there are two major categories: viral and non-viral vectors. Amongst the successful viral vectors, adenovirus and retrovirus, are the most commonly used vectors [39]. We had shown that adenovirus and retrovirus were used as a vector in 22% and 18.7% of the trials respectively. Herpes simplex virus and lentivirus were recent candidates in gene delivery used in 3.6% and 5.7% of the trials respectively.

Non-viral vectors are chemical and physical systems including: cationic liposomes and polymers, particle bombardment, electroporation, and ultrasound utilization. Non-viral vectors are less efficient than viral vectors, but their availability and cost-effectiveness are more important than the viral vectors [40]. Naked/plasmid DNA is used in 18% of the trials as a vector.

Delivering therapeutic genes into patients’ cells using efficient and safe vectors is considered a challenge that gene therapies are facing. Viral vectors may cause undesirable effects by stimulating the host’s immune system [41], and other problems exist, such as dose-related toxicity, pre-existing neutralizing antibodies, short-lived or insufficient transgene expression. Nonetheless, innovation is playing an important role in addressing this challenge. Reengineered adeno-associated virus (AAV) constitutes the next generation of AAV. For example, AAV2.5 has an antigenically distinct profile and can evade neutralizing antibodies against both AAV1 and AAV2 capsids [42].

### Market access of gene therapies

The innovation in research and development (R&D) depends on the incentives and obstacles set by the regulatory frameworks. A study of the French Pricing Committee in 2014 (Comité Economique des Produits de Santé, CEPS) concludes that ‘EU regulation matters at all stages of the innovation process from R&D to commercialisation.’ [43]

Regulators tend to speed up market access of innovative therapies. They are supporting medicines developers through early dialogues, priority medicine scheme (PRIME), a support dedicated to small and medium enterprises, supports dedicated to academia via Innovation Task Force and dedicated interactions, adaptive pathways [44], and accelerated pathways (authorisation under exceptional circumstances, conditional marketing authorisation, and accelerated assessment).

Therefore, the health products are expected to reach the market with immature evidence. This will be challenged by the payers. Health technology assessment (HTA) bodies/payers are increasingly scrutinising the incremental value of innovative therapies expected to have high prices. The first gene therapy approved in the EU in 2012, Glybera®, was not recommended by the French health authority (HAS) due to insufficient clinical benefit [45]. In Germany, the Federal Joint Committee (G-BA) decided that Glybera® has a ‘non-quantifiable’ added benefit [46]. The manufacturer of Glybera® was seeking a retail price of 53,000 euros per vial, which equates to 1.11 million euros per patient [47]. Payers were not equipped to assess and deal with the high prices of such advanced therapies. However, considering the important number of gene therapies in development, payers need to find new financing methods for these products.

A recent study demonstrated that if ATMPs, expected to cure, halt or slow the progression of many chronic and disabling diseases, reach the market successfully, they will have a huge budget impact and will likely threaten the sustainability of national health insurance. If the ATMPs will be able to cure all patients with Alzheimer disease, Parkinson disease, and heart failure, the budget impact will be respectively £72,132,071,000 [48]; £12,116,077,312 [49]; €348,144,683,200 [50].

Many authors have proposed several funding models for high-cost innovative therapies that mainly aim to share the risk between the manufacturer and the payer. Edlin et al. proposed to lease the payment by the ‘technology leasing reimbursement strategy’ [51]; the up-front payments are replaced by a stream of payments spread over the expected duration of benefit, subject to the technology delivering the claimed health benefit. Kleinke et al. proposed three financing models for addressing the cost crisis of innovative therapies: high-cost drug mortgages; high-cost drugs reinsurance; and high-cost drug patient rebates [52].

Managed entry agreements (MEA) may represent a solution for improving access of gene therapies. MEA are instruments used to reduce the impact of high prices and uncertainty, they are negotiated between payers and manufacturers. A maximum price is set for each new therapy and may vary more or less downwards by MEA [53]. Different schemes are implemented in many countries; these schemes range from simple financial schemes (e.g., discount, pay-back, budget cap, PVAs) to performance-based scheme [54], and may combine both to reach complex schemes. Many countries use these arrangements to enable broader access to high-cost medicines with high uncertainty regarding effectiveness, cost-effectiveness, or budget impact at the time of regulatory approval [54]. In the UK, financially based agreements constitute a preferred approach; in Italy, pay-for-performance arrangements are required for high cost oncology therapies; whereas payers in the US have a limited experience with innovative pricing [55].

MEAs may not be a sufficient solution for affordability challenges [56]. Innovative reimbursement mechanisms are required to ensure patient access to those innovative gene therapies, to obtain best value for money, and to ensure affordability. Parallel advice may help harmonize HTA and regulators’ perspectives, provide manufacturers’ recommendations to achieve market access for innovative gene therapies, and reduce the gap between payers and regulators.

## Conclusion

The gene therapy field has experienced ups and downs between 1989 until 2012. After two decades of dashed expectations, the first gene therapy was approved in the EU in 2012. This likely created a boost to this field, and the development of gene therapies increased prominently between 2012 and 2015. Gene therapies are considered today as very promising therapies to treat many chronic and disabling diseases that were previously untreatable. Manufacturers are investing more in this field, and an increasing number of products are under clinical development, mostly in early stages. Regulators are creating specific path for rapid access of those new therapies, providing hope for manufacturers, healthcare professionals, and patients. However, those accelerated regulatory pathways are associated with immature evidence that may be challenged by payers and will likely delay reimbursement. The large number of products in development represents a threat for health insurance system sustainability, especially with the public ones, if the pricing process remains unchanged, or if the gross domestic product growth remains flat in the Western world. Payers may consider revising their willingness to pay or the pricing rules for innovation if such products flow quickly onto the market. Paradoxically, investors may not achieve the expected return on investment if large numbers ofgene therapies reach the market, as as current high pharmaceutical prices may not be sustainable, and prices will likely drop. We expect hopeful success and major steps forward for the gene therapy field in the upcoming years, based on current therapies in development and accumulated know-how in this field over the last decade.

## Supplementary Material

Supplementary Table
