# Supplementary material for: Gene therapies development: slow progress and promising prospect
Source: J Mark Access Health Policy. 2017 Jan 3;5(1):1265293. doi: 10.1080/20016689.2017.1265293 (PMC5328344; doi:10.1080/20016689.2017.1265293)
Supplement: Supplementary Table [file zjma_a_1265293_sm1395.docx]

Supplementary materials:

Gene therapy consists of the delivery of DNA into human cells. It can be accomplished by several methods, summarized below. The two major classes of methods are those that use recombinant viruses (sometimes called biological nanoparticles or viral vectors) and those that use naked DNA or DNA complexes (non-viral methods).

Table S1: the most commonly used gene vectors:

|  | Vector | Description |
| --- | --- | --- |
| Viral vectors | Adenovirus | Icosahedric, non- enveloped, genome of 36 kb, non-integrative |
|  | Retrovirus | Enveloped, single-stranded positive-sense RNA virus , integrative in proliferative cells |
|  | Lentivirus | Enveloped, single-stranded positive-sense RNA virus, Integrative in proliferative and quiescent cells |
|  | Adeno-associated virus (AAV) | Icosahedric, non- enveloped, single-stranded DNA, genome of 4.7 kb,integrative |
|  | Vaccinia virus | Large, complex, enveloped virus belonging to the poxvirus family, genome approximately 190 kbp in length |
|  | Herpes simplex virus | Neurotropic DNA virus , mostly examined for gene transfer in the nervous system, large genome (152 Kb) |
|  | Poxvirus | Large, complex, enveloped virus with a double DNA chain |
| Non-viral vectors | Nude DNA | Direct injection of plasmidial DNA into target cell (non-complexed to any particle) |
|  | Lipofection (liposome transfection) | Injecting genetic material into a cell via liposomes, which are vesicles made of a phospholipid bilayer. |
|  | RNA transfer | Small RNAs are molecules containing 75 to 95 nucleotides. RNA can be transfected into cells to transiently express its coded protein |
